# Optoelectrical Properties of Transparent Conductive Films Fabricated with Ag Nanoparticle-Suspended Emulsion under Various Formulations and Coating Conditions

**DOI:** 10.3390/nano13071191

**Published:** 2023-03-27

**Authors:** Seong Hwan Kim, Geunyeop Park, Kyu-Byung Kim, Yong-Woo Shin, Hyun Wook Jung

**Affiliations:** 1 Department of Chemical and Biological Engineering, Korea University, Seoul 02841, Republic of Korea; 2 Dof Inc., Hwaseong-si 18468, Republic of Korea

**Keywords:** transparent conductive films, Ag nanoparticles, emulsion coating, optoelectrical properties, droplet size, self-assembled network

## Abstract

Transparent conductive films (TCFs) were fabricated through bar-coating with a water-in-toluene emulsion containing Ag nanoparticles (AgNPs). Morphological changes in the self-assembled TCF networks under different emulsion formulations and coating conditions and the corresponding optoelectrical properties were investigated. In preparing various emulsions, the concentration of AgNPs and the water weight fraction were important factors for determining the size of the water droplets, which plays a decisive role in controlling the optoelectrical properties of the TCFs affected by open cells and conductive lines. An increased concentration of AgNPs and decreased water weight fraction resulted in a decreased droplet size, thus altering the optoelectrical properties. The coating conditions, such as coating thickness and drying temperature, changed the degree of water droplet coalescence due to different emulsion drying rates, which also affected the final self-assembled network structure and optoelectrical properties of the TCFs. Systematically controlling various material and process conditions, we explored a coating strategy to enhance the optoelectrical properties of TCFs, resulting in an achieved transmittance of 86 ± 0.2%, a haze of 4 ± 0.2%, and a sheet resistance of 35 ± 2.8 Ω/□. TCFs with such optimal properties can be applied to touch screen fields.

## 1. Introduction

Transparent conductive films (TCFs) are optically transparent and electrically conductive. They have been widely applied in industrial fields such as touch screens [1,2,3], organic solar photovoltaics [4], organic light-emitting diodes (OLEDs) [5,6,7], liquid crystal displays (LCDs) [8,9,10,11], and transparent conducting heaters [12]. Indium tin oxide (ITO), which has a high transmission and low electrical resistivity, is a popular material for fabricating TCFs [13]. However, the critical disadvantages of ITO, such as brittleness and high cost, are actively driving the development of alternative materials for applications in flexible electronic devices [14,15,16]. For this purpose, metallic nanostructures, including metal thin films, metal nanogrids, and metal nanowire networks, are fascinating alternatives because of their unique optoelectronic properties. In particular, metal (for example, Ag) nanowire networks have been considered excellent candidates because they exhibit a better performance compared to ITO [17]. In addition, TCFs containing Ag nanowires can be effectively fabricated at low cost via a solution-based roll-to-roll process. However, the high junction resistance resulting from the weak contact between Ag nanowires and surface roughness is the main drawback that requires a solution [18,19]. In this situation, Ag nanoparticles (AgNPs) are used to produce Ag-coated films with excellent optical and electrical performance by efficiently decreasing sheet resistance through tight junctions between particles [20,21].

TCFs with AgNPs are typically fabricated through the self-assembly of nanoparticles. After the thin-film coating process with a AgNP-suspended emulsion at atmospheric pressure, random mesh-like network structures composed of AgNPs are formed via spontaneous self-assembly as evaporation proceeds. The driving force for network structure formation originates from the coffee ring effect [22], which is induced by the different evaporation rates of the continuous phase and dispersed phase solvents. Through this operation, both the desired optical transparency and electrical conductivity can be achieved using open cells surrounded with AgNPs and conductive lines composed of AgNPs, respectively. Therefore, network structures can be achieved economically through this inexpensive maskless coating method. This is a promising film fabrication process that ensures superb film properties during mass production [23].

The droplet size of the dispersed phase in the emulsion is the most important factor for generating network structures because AgNPs are deposited along the contact lines of the dispersed phase droplets. This is directly related to the optical and electrical performance of the TCFs.

To determine emulsion stability and droplet size, numerous factors can be used, including the weight ratio of the two solvents [24], nanoparticle concentration [25], structure of surfactants [26], stirring intensity [27], mixing time [28], and mixing temperature [27,29]. In addition, coating conditions, such as coating thickness and drying temperature, are important because they can affect the coalescence degree of droplets and the corresponding final structures of the TCFs by regulating the total drying time. Therefore, the establishment of proper emulsion preparation and coating conditions is indispensable for the optimal performance of TCFs.

Thus far, AgNPs have been mainly studied in combination with other materials, such as graphene nanosheets [30], Ag nanowires [20], and carbon nanomaterials [31]. The performance of TCFs fabricated with only AgNPs has been examined under limited emulsion-formulating conditions, such as the size and shape of AgNPs [32]. Thus, it is important to systematically correlate the overall coating process conditions with the structure and properties of the AgNP-based TCFs.

In this study, the effects of the emulsion formulation and drying conditions on the final optical and electrical properties of TCFs were investigated. Water-in-toluene emulsions were prepared by changing the concentration of AgNPs and the water fraction based on a constant solvent weight. Optical microscopy was used to observe the dispersed phase droplets, that is, water droplets, in various formulations. In the bar-coating stage of emulsions, thin coating films of different thicknesses were produced using different bars and drying temperatures within the convection oven. The final TCF structures were observed using scanning electron microscopy (SEM). The optical properties, such as haze and total transmittance, were measured using a haze meter, and the electrical properties, such as sheet resistance, were measured with a low resistivity meter that had a four-point probe.

## 2. Materials and Experimental Methods

### 2.1. Preparation of Water-in-Toluene Emulsions and Transparent Conductive Films

#### 2.1.1. Water-in-Toluene Emulsions

First, AgNPs in the range 0.519–1.038 g (AGFA, Mortsel, Belgium, D = 30–80 nm) were added to a mixture of 0.026 g of Span 60 (DUKSAN, Ansan-si, Korea), 2.14 g of cyclohexanone (DUKSAN, Ansan-si, Korea), 0.057 g of BYK 410 (BYK, Wesel, Germany), and 23.03 g of toluene (DAEJUNG, Siheung-si, Korea). Subsequently, an ultrasonic homogenizer (UW 2070, BANDELIN, Berlin, Germany) was used to disperse the AgNPs twice for 30 s each. After the dispersion step, 0.08 g of APS, which is a mixture of hexamethoxymethylmelamine (Sigma-Aldrich, St. Louis, MO, USA) and p-toluenesulfonic acid (Sigma-Aldrich, St. Louis, MO, USA), 0.86 g of Dispo, which is a mixture of 2-amino-1-butanol (Sigma-Aldrich, St. Louis, MO, USA) and polyether siloxane (Sigma-Aldrich, St. Louis, MO, USA), and 23.11 g of DI water were added. Ultrasonic homogenization was conducted three times, each time for 30 s, for emulsification.

Next, various emulsions were formed with different water weight fractions (based on the total solvent weight) in the range 0.4–0.55 under conditions where the total solvent and other substances were kept constant. The appropriate concentration of AgNPs was selected based on experimental results, such as network structures and optoelectrical properties. The dispersion and emulsification processes were optimized in the same manner. Through these procedures, water(dispersed phase)-in-toluene(continuous phase) emulsions were produced, and the formulations of the emulsions used in this study are listed in Table 1.

#### 2.1.2. Transparent Conductive Films

Polyethylene terephthalate (PET) films (Higashiyama Film, Nagoya, Japan) of 18 cm width and 27 cm height were coated with primer, which is a mixture of methyl ethyl ketone (DUKSAN, Ansan-si, Korea), methyl isobutyl ketone (DUKSAN, Ansan-si, Korea), hydroxy dimethyl acetophenone (Sigma-Aldrich, St. Louis, MO, USA), and polyethylene glycol diacrylate (Sigma-Aldrich, St. Louis, MO, USA). The 6.86 μm-thick primer coating was applied using a bar-coater (DAO-CO02-SERVO, DAO Technology, Uiwang-si, Korea) at a speed of 167 mm/s to enhance the emulsion coating. The film was then dried at 50 °C for 1 min in a convection oven (OF-02GW, JeioTech, Daejeon, Korea) and UV-cured using a UV-curing benchtop conveyor (LC6B, Fusion UV Systems, Inc., Gaithersburg, MD, USA). Next, the primer-coated PET films were maintained at room temperature for 24 h for sufficient aging.

The coating films using the emulsions listed in Table 1 were fabricated via bar-coating at a speed of 167 mm/s with a wet thickness of 27.4 μm. The films were then dried at 50 °C for 1–2 min and thermally cured at 150 °C for 2 min.

After determining the optimal emulsion formulation, the optimal wet coating thickness and drying temperature were sequentially investigated in the 27.4–96.0 μm thickness range (using the coating bars), and in the temperature range between 30 and 90 °C (controlled using the convection oven [OF-02GW, JeioTech, Daejeon, Korea]). The coating conditions are presented in detail in Table 2.

#### 2.1.3. Formation of Self-Assembled Network Structures in TCFs

**Figure 1:**
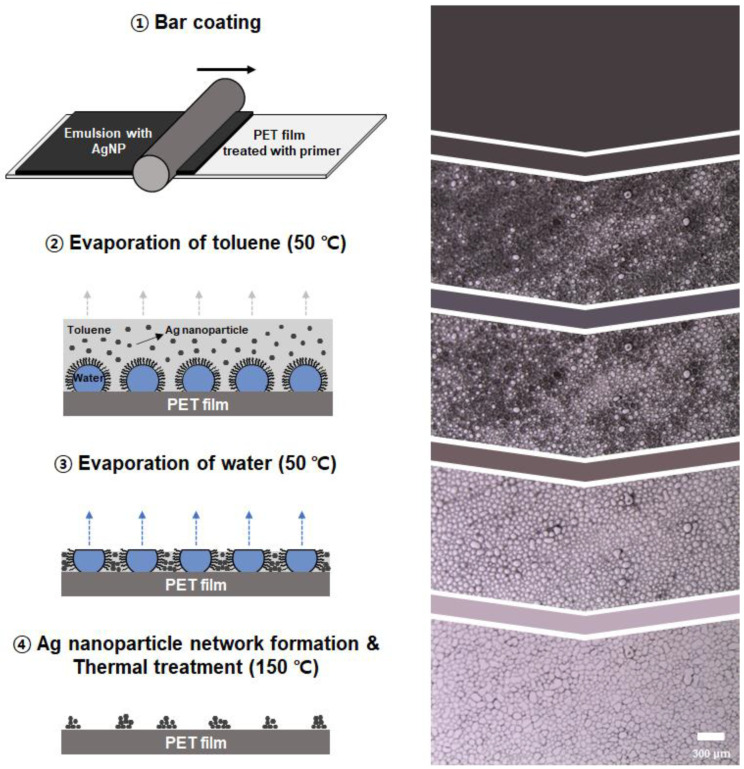
Schematic illustration for manufacturing TCFs and stepwise top-view images of the emulsions.

The overall process of TCF fabrication is illustrated in Figure 1. First, a conductive water-in-toluene emulsion with AgNPs was coated onto a solid substrate, that is, a primer-coated PET film, via bar-coating. After the coating stage, water droplets with a higher density were distributed on the substrate surface in the form of isolated islands. The AgNPs, which were dispersed in the toluene phase, were not allowed to penetrate the water droplets and were thus captured on the surfaces of the droplets. As more volatile toluene evaporated, the water droplets separated by a continuous phase began to form polygonal shapes. The spaces separating the droplets became thinner, and the capillary pressure caused by drying led to droplet coalescence as evaporation proceeded [33]. Finally, both toluene and water completely evaporated, and the AgNPs spontaneously self-assembled into a microsized network structure. Subsequently, a high-temperature thermal treatment was conducted to strengthen the network structures. In this study, the effect of the material and process conditions—such as the concentration of AgNPs, water fraction, coating thickness, and drying temperature—on the final network structures of AgNPs was scrutinized based on the TCF manufacturing mechanism.

### 2.2. Characterization of Emulsions and TCFs

#### 2.2.1. Observation of Water Droplets in Water-in-Toluene Emulsions

The size of the water droplets (dispersed phase) in the emulsions was significantly related to the final network structures. The emulsions were slightly diluted with toluene because of the very dark color of the AgNPs. The amount of toluene added was determined based on the consideration of not breaking the water droplets. Water droplets in the water-in-toluene emulsions with different AgNP concentrations and water weight fractions were observed using an optical microscope (LILY MCX500, Micros Austria, Gewerbezone, Austria) after the same time from loading emulsions.

#### 2.2.2. Optical and Electrical Properties of TCFs

The total transmittance and haze of the TCFs were measured using a haze meter (NDH 5000, Nippon Denshoku, Tokyo, Japan). Total transmittance is the fraction of incident light that is transmitted in the range of visible light, and haze is the ratio of sample diffusion to total transmittance. The enhancement of the optical properties represents increased total transmittance and decreased haze. Having conducted a total of 10 measurements at various positions on TCFs, the average values of these parameters were plotted. The morphologies of the TCFs were observed using SEM (S-4800, Hitachi, Tokyo, Japan) to relate the optical properties to the network structure.

The sheet resistance, which is the bulk resistivity divided by the sheet thickness, was measured using a low resistivity meter with a four-point probe (MCP-T610, Mitsubishi Chemical, Tokyo, Japan). Highly conductive films have low sheet resistance values. The sheet resistance at multiple positions on TCFs was measured 10 times, and the resulting average values were plotted.

## 3. Results and Discussion

### 3.1. Effect of Emulsion Formulation

#### 3.1.1. Concentration of AgNPs

**Figure 2:**
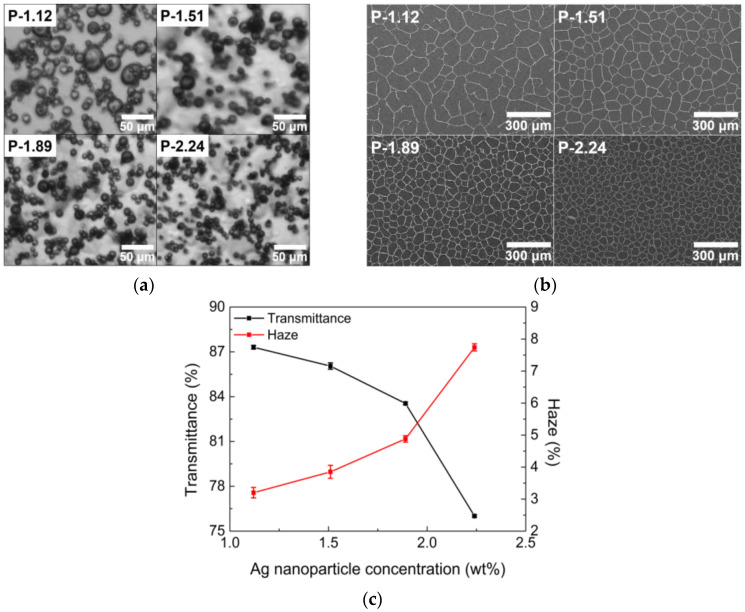
(**a**) Optical images of droplets in emulsion, (**b**) SEM images of TCFs, and (**c**) optical properties of TCFs with different concentration of AgNPs.

An increase in the concentration of nanoparticles usually enhances emulsion stability [25,34]. When Ag nanoparticles were dispersed in the emulsion, the interfaces between the water and toluene in the emulsion became covered with AgNPs and a particle layer was formed around each water droplet, which prevented the coalescence of water droplets and stabilized the emulsion. When additional nanoparticles were added, large water droplets were broken down into smaller ones because a greater number of AgNPs could then be adsorbed on the droplet surfaces. Therefore, an increase in the nanoparticle concentration can effectively reduce the average size of the emulsion droplets [35,36], as shown in Figure 2a. In addition, Figure 2b shows that the size of open cells surrounded by AgNPs decreases with an increase in the concentration of AgNPs. The change in cell size can directly affect the optoelectrical properties of the TCFs. The decrease in open cell size leads to the degradation of the optical properties of the TCFs; that is, the transmittance decreases and haze increases, as displayed in Figure 2c, because the open cell size is normally responsible for the optical properties.

**Figure 3:**
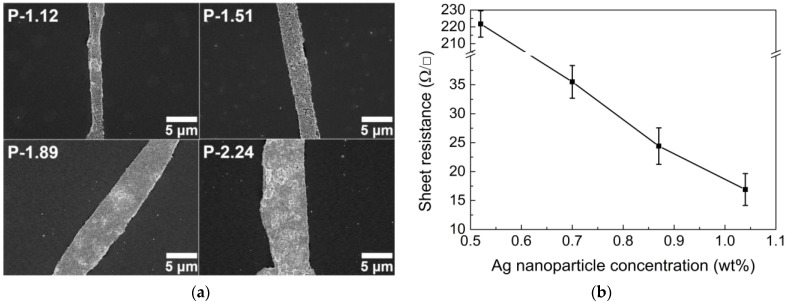
(**a**) SEM images of conductive lines in TCFs and (**b**) sheet resistance with different concentration of Ag nanoparticles.

The conductive lines formed with AgNPs are directly responsible for the electrical properties, that is, the sheet resistance. As more AgNPs are added under the given conditions of P-1.12 to P-2.24, the conductive lines become wider because of the dense deposits, as shown in Figure 3a. Note that the conductive lines become wider with an increase in the AgNP concentration, even if the number of smaller droplets increases. Therefore, the probability of current leakage is suppressed with increasing AgNPs, resulting in a decrease in the sheet resistance (Figure 3b). In addition, the extremely high sheet resistance of the P-1.12 sample is caused by both the thin conductive lines and the incomplete network structure of the TCFs, owing to the insufficient concentration of AgNPs.

As a result of the optoelectrical properties, the concentration of AgNPs was found to be in an appropriate range (between 1.51 wt% and 1.89 wt%) because the P-1.12 and P-2.24 samples were not recommended owing to their high sheet resistance and poor optical properties, respectively. The optimal concentration of AgNPs was chosen to be 1.51 wt%, guaranteeing better optical properties.

#### 3.1.2. Water Weight Fraction

**Figure 4:**
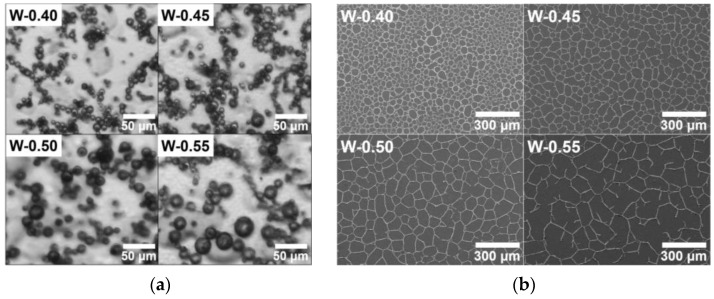

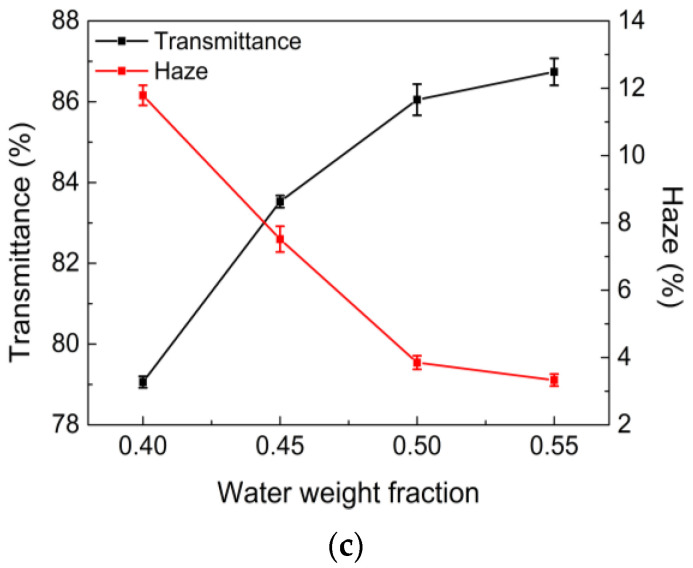
(**a**) Optical images of droplets in emulsion, (**b**) SEM images of TCFs, and (**c**) optical properties of TCFs with different water weight fractions.

An increased water fraction in the emulsion gives rise to larger water droplets existing in the form of isolated islands [24]. Figure 4a,b show the increased droplet size and corresponding larger cell size as the water fraction increases. The optical properties are enhanced as the water fraction increases owing to the large size of open cells in the network structure.

**Figure 5:**
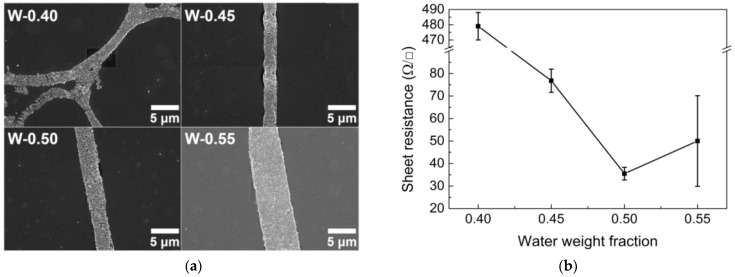
(**a**) SEM images of conductive lines in TCFs and (**b**) sheet resistance under different water weight fraction conditions.

As the droplet size increases, the total surface area of the droplets with adsorbed AgNPs tends to decrease. Therefore, the conductive lines become wider because of the constant concentration of AgNPs, resulting in a decrease in sheet resistance (Figure 5a,b). However, in the W-0.55 case, the sheet resistance is rather high because the network is not tightly formed. The particularly high sheet resistance of the W-40 sample is due to the large number of small break-ups of conductive lines, which is caused by the relative insufficiency of AgNPs owing to the increase in the total surface area of the droplets, as shown in Figure 5a. Based on the results shown in Figure 4 and Figure 5, W-0.50 was considered the optimal water fraction, providing good optical properties and low sheet resistance.

### 3.2. Effect of Coating Conditions

#### 3.2.1. Coating Thickness

**Figure 6:**
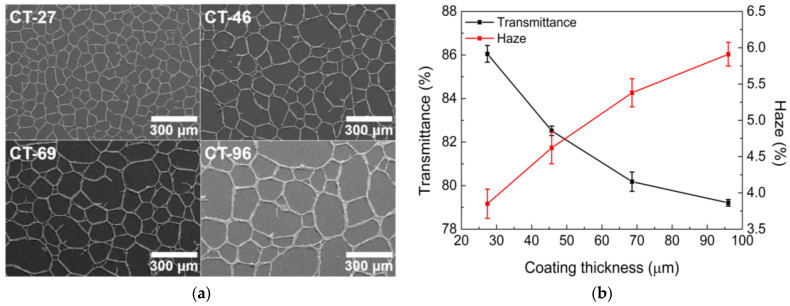
(**a**) SEM images and (**b**) optical properties of TCFs under different coating thickness conditions.

After determining the optimal emulsion formulation, the wet coating thickness was controlled by changing the diameter of the bar used in the bar-coating process. As the coating thickness increases, the emulsion load increases, and more time is required for drying. Therefore, it is more likely for water droplets to coalesce into larger droplets as drying continues, which increases the cell size (Figure 6a). However, the optical properties degrade even when the size of the open cell is increased (Figure 6b). For the determination of the optical properties, as the coating thickness increases, the increased width of the overall conductive lines becomes a more influential factor than the size of the open cells.

**Figure 7:**
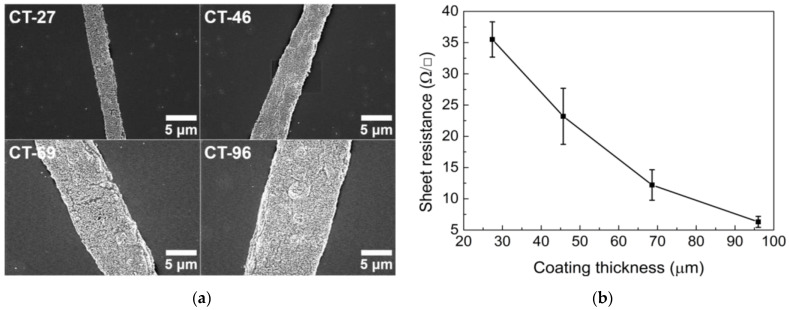
(**a**) SEM images of conductive lines in TCFs and (**b**) sheet resistance under different coating thickness conditions.

A thicker coating indicates an increase in the loading amount of the emulsion; accordingly, there are more AgNPs consisting of conductive lines, making the conductive lines wider and denser, as portrayed in Figure 7a. There is little possibility of current leakage as the coating thickness increases. Therefore, the sheet resistance decreases as the coating thickness increases (Figure 7b).

There is a trade-off between the optical and electrical properties with increasing coating thickness, which means that the optimal coating thickness of TCFs can be determined, depending on the required properties for various applications. The required performance of TCFs for touch screen applications should satisfy a sheet resistance of 30–40 Ω/□ and a transmittance of 85% or more. Therefore, it is confirmed that a coating thickness of 27.4 μm is optimal. Notably, in a network structure with a very thin wet coating (thinner than 27.4 μm, for example), it is expected for a large number of break-ups to occur because of the extremely high drying rate.

#### 3.2.2. Drying Temperature

The drying temperature was controlled using a forced convection oven. The drying temperature predominantly affected the drying rate of the emulsion. The lower the drying rate, the greater the coalescence of water droplets in the emulsion becomes, resulting in larger open-cell regions during drying.

**Figure 8:**
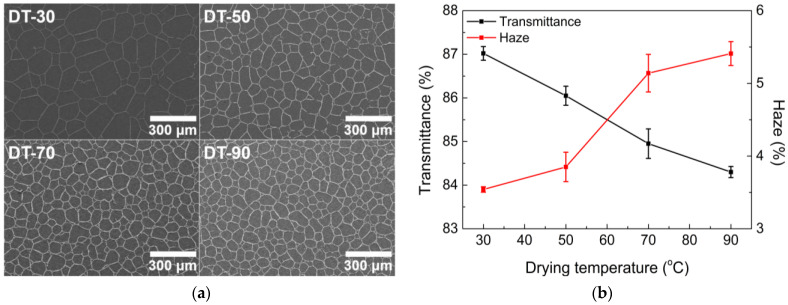
(**a**) SEM images and (**b**) optical properties of TCFs under different drying temperature conditions.

**Figure 9:**
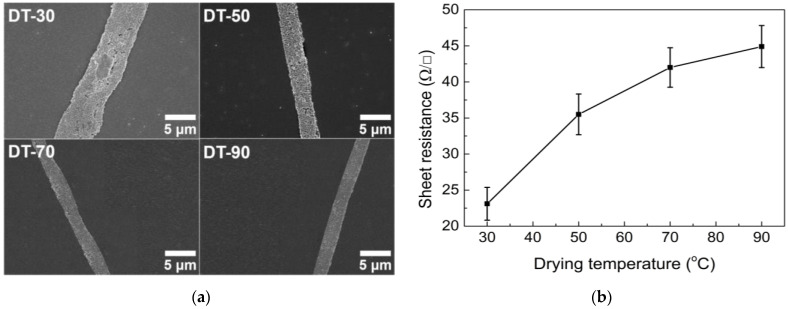
(**a**) SEM images of conductive lines in TCFs and (**b**) sheet resistance under different drying temperature conditions.

When the drying temperature is low (for example, 30 °C), the drying speed is slow enough to easily induce the coalescence of water droplets. Consequently, the size of open cells becomes relatively large (Figure 8a). As the drying temperature is gradually increased to 70 and then to 90 °C, cell size decreases because rapid drying prevents the coalescence of droplets. Therefore, the transmittance decreases and haze increases with increasing drying temperature (Figure 8b). A higher drying temperature induces a smaller open cell size, reducing the width of the conductive lines under a constant AgNP concentration (Figure 9a). Therefore, the increased sheet resistance was measured at high drying temperatures because of the higher possibility of conductive line break-ups (Figure 9b). Note that drying temperatures over 70 °C do not significantly change the sheet resistance because the emulsions dry very rapidly at such temperatures.

Based on the above results, a low-temperature range between 30 and 50 °C was found to be an appropriate drying temperature to produce TCFs with good optoelectrical properties. Thus, 50 °C was chosen as the optimal drying temperature because, at this temperature, the overall network structure was formed more closely, reducing the probability of critical break-ups, which can cause the malfunctioning of the final products.

## 4. Conclusions

Transparent conductive films (TCFs) were fabricated using a water-in-toluene emulsion containing AgNPs. The concentration of AgNPs and the water weight fraction were varied when preparing emulsions, and the coating thickness and drying temperature were controlled during the bar-coating process. The droplets in the emulsion were observed using an optical microscope, and the morphology of the self-assembled TCFs was observed using SEM. The optical and electrical properties were measured using a haze meter and low resistivity meter with a four-point probe, respectively. In some cases, the optical properties were determined by the size of the open cell and the width of the conductive lines composed of AgNPs. The electrical properties were affected by the width of the conductive lines and overall degree of self-assembled network connectivity. The water droplet size was determined to be the most important factor in preparing emulsions because it directly affected the size of the open cell in the TCFs. When the concentration of AgNPs was increased, the droplet size decreased, resulting in degraded optical and enhanced electrical properties. With a fixed concentration of AgNPs, we varied the water weight fraction and observed a decrease in the droplet size at low water weight fractions. This results in the degradation of the optoelectrical properties, contrary to the case with a high concentration of AgNPs. When the drying conditions were changed, the optoelectrical properties of the TCFs changed even when the same emulsion was applied. When the films were thickly coated, the optical properties degraded and the electrical properties were enhanced. As the drying temperature decreased, the optoelectrical properties improved. However, drying at too low a temperature may cause significant defects in TCFs. In this study, the optimal conditions throughout the TCF fabrication process were identified. Based on the experiments, we concluded that a concentration of AgNPs in the range of 1.51 wt%, a water weight fraction of 0.50, a coating thickness of 27 μm, and a drying temperature of 50 °C were the optimal conditions for fabricating TCFs. Under these conditions, the production of TCFs with optimal optoelectrical properties is expected to be advantageous to various industries, such as the manufacturing of displays and touch screens. In order to manufacture self-assembled TCFs that exhibit improved stability and flexibility, while guaranteeing transparency, it is crucial to well establish coating and drying conditions that are customized for the specific display products.

## Figures and Tables

**Table 1:** Emulsion formulations with various AgNP concentrations and water weight fractions. (P stands for the AgNP concentration with respect to total solvent weight; W stands for the water weight fraction based on the total solvent weight). Other additives for preparing emulsions, such as Span 60, cyclohexanone, and APS, are not listed in the table.

Sample	AgNP (g)	Toluene (g)	DI Water (g)	Water Weight Fraction to the Total Solvent Weight
P-1.12	0.52	23.03	23.11	0.5
P-1.51	0.70	23.03	23.11	0.5
P-1.89	0.87	23.03	23.11	0.5
P-2.24	1.04	23.03	23.11	0.5
W-0.40	0.70	27.68	18.46	0.40
W-0.45	0.70	25.38	20.76	0.45
W-0.50	0.70	23.03	23.11	0.50
W-0.55	0.70	20.76	25.38	0.55

**Table 2:** Coating-drying conditions of the optimally formulated emulsion. (CT stands for the coating thickness; DT stands for the drying temperature.)

Sample	Coating Thickness (μm)	Drying Temperature (°C)
CT-27	27.4	50
CT-46	45.7	50
CT-69	68.6	50
CT-96	96.0	50
DT-30	27.4	30
DT-50	27.4	50
DT-70	27.4	70
DT-90	27.4	90

## Data Availability

Not applicable.
